# Species identification of adult African blowflies (Diptera: Calliphoridae) of forensic importance

**DOI:** 10.1007/s00414-017-1654-y

**Published:** 2017-08-28

**Authors:** Lena Lutz, Kirstin A. Williams, Martin H. Villet, Mfon Ekanem, Krzysztof Szpila

**Affiliations:** 1 Institute of Forensic Medicine, Goethe-University Frankfurt, Kennedyallee 104, D-60596 Frankfurt am Main, Germany; 2 Entomology Department, Durban Natural Science Museum, Durban, South Africa; 3 Southern African Forensic Entomology Research Laboratory, Department of Zoology and Entomology, Rhodes University, Grahamstown, South Africa; 4 Department of Zoology, University of Uyo, Uyo, Nigeria; 5 Faculty of Biology and Environmental Protection, Nicolaus Copernicus University, Lwowska 1, 87-100 Toruń, Poland

**Keywords:** Calliphoridae, Blowflies, Species identification, Africa, Forensic entomology

## Abstract

Necrophagous blowflies can provide an excellent source of evidence for forensic entomologists and are also relevant to problems in public health, medicine, and animal health. However, access to useful information about these blowflies is constrained by the need to correctly identify the flies, and the poor availability of reliable, accessible identification tools is a serious obstacle to the development of forensic entomology in the majority of African countries. In response to this need, a high-quality key to the adults of all species of forensically relevant blowflies of Africa has been prepared, drawing on high-quality entomological materials and modern focus-stacking photomicroscopy. This new key can be easily applied by investigators inexperienced in the taxonomy of blowflies and is made available through a highly accessible online platform. Problematic diagnostic characters used in previous keys are discussed.

**Electronic supplementary material:**

The online version of this article (doi:10.1007/s00414-017-1654-y) contains supplementary material, which is available to authorized users.

## Introduction

The correct identification of necrophagous blowflies is pivotal to their use as evidence in forensic entomology [1] and is also central to addressing the problems that these flies pose for public health, medicine, and animal health [2, 3]. Although superficially similar in appearance, the various species differ in their developmental biology, ecology, and behaviour and may variously be pests in meat and fish markets [4], causes of human and animal myiasis [3], or carriers of microbial pathogens [4, 5]. Correct identifications provide access to relevant information about these blowflies, especially the crucial studies of development [1].

Africa is home to over a dozen species of necrophagous blowflies, several of which are found on other continents too [1, 6]. The blowflies (family Calliphoridae) in their traditional, broad taxonomic concept were postulated to be a paraphyletic taxon [7], which was confirmed by subsequent molecular studies [8–11]. All species of forensically important blowflies are grouped in four subfamilies in the sense of the classification proposed by Rognes [7, 12]. Necrophagous blowfly faunas have been intensively studied in Australasia, Europe, East and South Asia, Middle East, and North and South America, resulting in a variety of published checklists, keys, and taxonomic monographs [12–22].

However, the blowfly faunas of some large geographical regions, like the Afrotropics (except for Namibia and the Republic of South Africa), remain poorly studied [3, 23, 24]. Except for the genus *Chrysomya*, keys, if available, do not cover the fauna of the whole continent and often are poorly illustrated [23–25]. This situation hampers the broad application of insects for medico-legal purposes due to a lack of proper tools for species identification in some African countries. Additionally, there is a widespread lack of access to printed material in Africa, but the high penetration of internet-enabled mobile phone technology in Africa is making access to Health and open-access electronic media an increasingly practical alternative [26, 27].

The aim of the present paper is to deliver a high quality, open-access key for identification of the adults of all species of forensically relevant blowflies known from Africa. The main intention was to prepare a species identification tool that will be easily applicable to investigators inexperienced in blowfly taxonomy. This task was achieved thanks to the application of modern focus-stacking photomicroscopy [28, 29] and the use of suitably preserved entomological material. The authors hope that this publication will be a further milestone toward advancing the application of forensic entomology in African countries.

## Material and methods

The key contains 15 species verified as necrophagous and one obligately parasitic species (*Chrysomya bezziana*, Old World screwworm) and includes nomenclatural synonyms that are important in accessing relevant biological literature. Most of the specimens used to illustrate characters were collected personally by the authors. Specimens of *Chrysomya inclinata*, *Chrysomya laxifrons*, *Chrysomya chloropyga*, and *Lucilia infernalis* were collected by Dr. Amnon Freidberg (Tel Aviv University) during field trips to Kenya and Malawi. An additional large series of specimens of *Ch. chloropyga* was donated by Dr. Marise Heyns (University of Cape Town). Specimens of *Ch. bezziana* were provided by Dr. April H. Wardhana (IRCVC, Indonesia).

The preparation of image-stacking was done using an M205C Leica Stereomicroscope with an integrated high-resolution Leica DFC495 digital camera and associated software (Leica Application Suite 4.4.0). For the compilation of final pictures, 30–35 images were stacked. Characters used in the key were mostly compiled from Zumpt [23], Rognes [12, 16], Wallman [18], Rognes and Paterson [30], Kurahashi and Kirk-Spriggs [24], Szpila [31], Irish et al. [25], Williams and Villet [32], and Akbarzadeh et al. [22]. The terminology follows Rognes [12], and all morphological details were clearly marked and abbreviated on the figures.

Distributional data were restricted to the African mainland and follow Zumpt [23], Pont [6], Erzinçlioğlu [33], Verves [19, 34], Ekanem and Dike [35], Irish et al. [25; text, not maps], and Taleb et al. [36]. Many locality records reported in the listed references need verification and should be treated with caution. Former records referring to Sudan were not revised into Sudan and South Sudan, because detailed verification of the collection sites was not generally possible. New distribution records for some species for specific countries resulted from the inspection of collections by the authors (KwaZulu-Natal Museum [Durban], National Museum [Bloemfontein], Iziko Museum—South Africa [Cape Town], Natural History Museum of Denmark [Copenhagen]). These new records are indicated in the key by underlining the names of the countries concerned.

## Results

### Key to the African blowflies of forensic importance

**Fig. 1:**
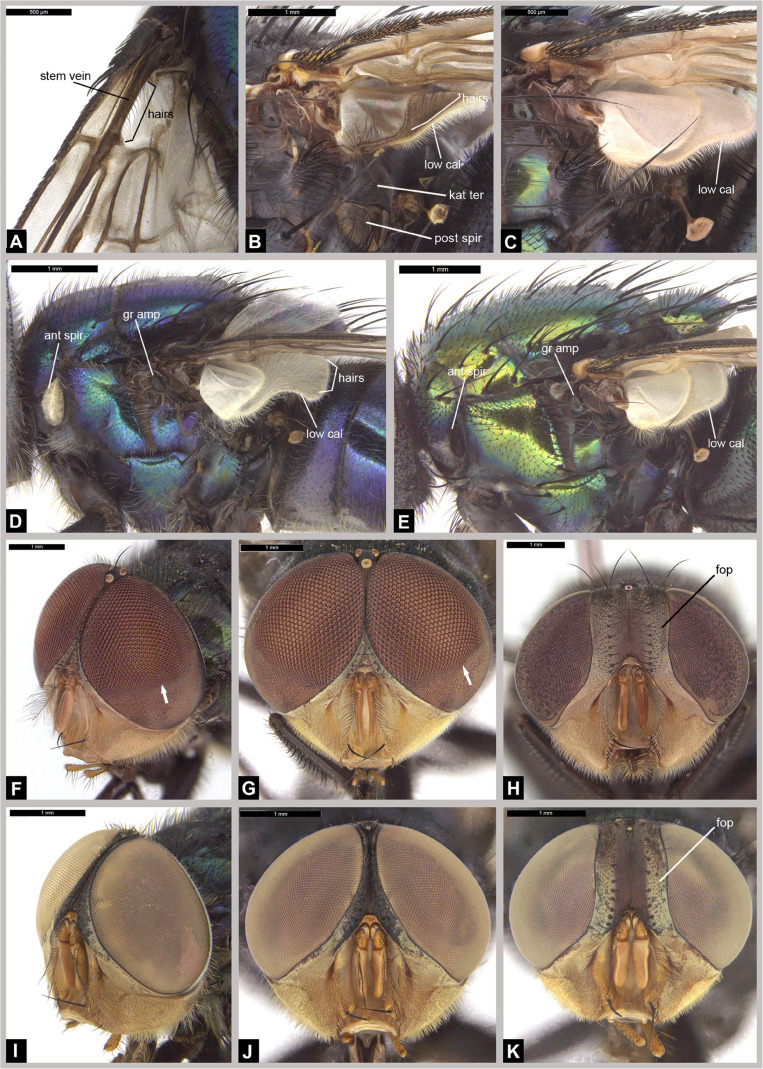
Morphology of African blowflies of forensic importance. **a** *Ch. albiceps*, basal part of wing, stem vein. **b** *C. vicina*, thorax, upper and lower calypters. **c** *L. sericata*, thorax, upper and lower calypters. **d** *Ch. albiceps*, thorax, lateral view. **e** *L. sericata*, thorax, lateral view. **f** *Ch. megacephala*, male, head, fronto-lateral view, arrow points at demarcation line between small and large eye facets. **g** *Ch. megacephala*, male, head, frontal view, arrow points at demarcation line between small and large eye facets. **h** *Ch. megacephala* female, head, frontal view. **i** *Ch. bezziana*, male, head, fronto-lateral view. **j** *Ch. bezziana*, male, head, frontal view. **k** *Ch. bezziana,* female, head, frontal view. *ant spir* anterior spiracle, *fop* fronto-orbital plate, *gr amp* greater ampulla, *kat ter* katatergite, *low cal* lower calypter, *post spir* posterior spiracle

**Fig. 2:**
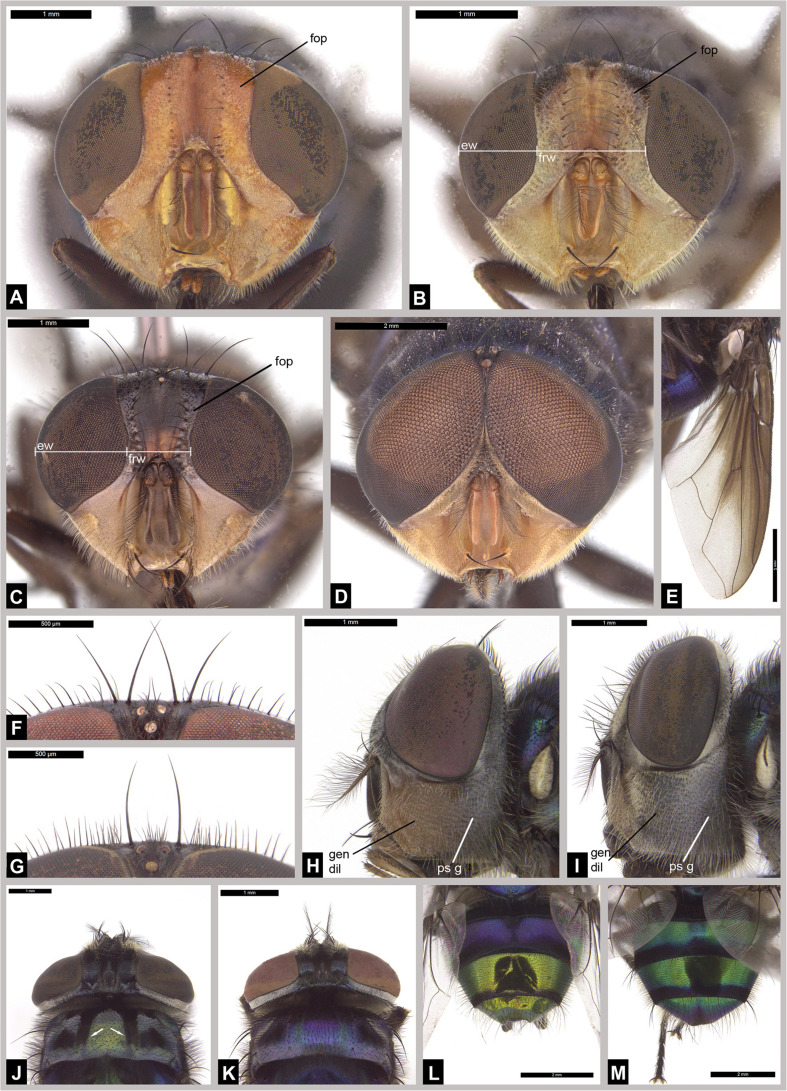
Morphology of African blowflies of forensic importance. **a** *Ch. marginalis*, female, head, frontal view. **b** *Ch. laxifrons*, female, head, frontal view. **c** *Ch. inclinata*, female, head, frontal view. **d** *Ch. marginalis*, male, head, frontal view. **e** *L. infernalis*, wing. **f** *Ch. albiceps*, male, head, top of frons. **g** *Ch. putoria*, male, head, top of frons. **h** *Ch. albiceps*, head, lateral view. **i** *Ch. chloropyga*, head, lateral view. **j** *Ch. chloropyga*, thorax, dorsal view. **k** *Ch. putoria*, thorax, dorsal view. **l** *Ch. chloropyga*, abdomen, dorsal view. **m** *Ch. putoria*, abdomen, dorsal view. *ew* eye width, *fop* fronto-orbital plate, *frw* frons width, gen *dil* genal dilation, *ps g* post gena

**Fig. 3:**
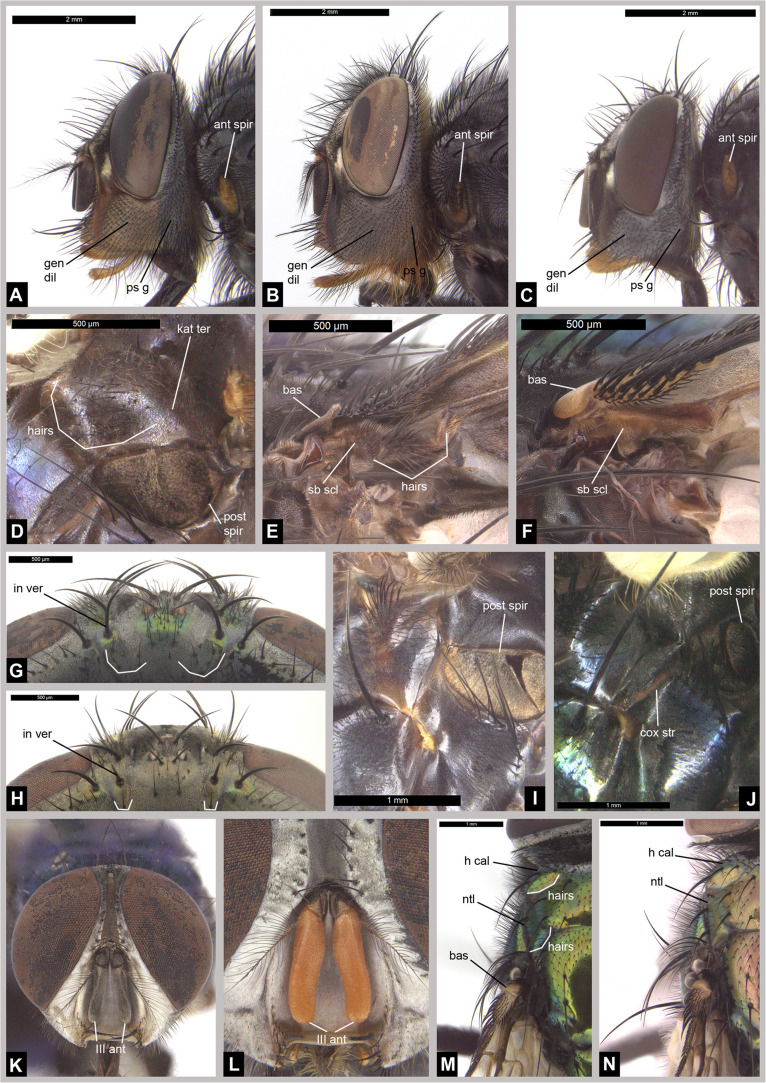
Morphology of African blowflies of forensic importance. **a** *C. vicina*, head, lateral view. **b** *C. vomitoria*, head, lateral view. **c** *C. croceipalpis*, head, lateral view. **d** *H. fernandica*, thorax, posterior part, lateral view. **e** *L. infernalis*, thorax, base of wing, lateral view. **f** *L. sericata*, thorax, base of wing, lateral view. **g** *L. sericata*, head, posterior view. **h** *L. cuprina*, head, posterior view. **i** *C. vicina*, thorax, posterior part, lateral view. **j** *L. sericata*, thorax, posterior part, lateral view. **k** *H. fernandica*, head, anterior view. **l** *H. pulchra*, head, antennae. **m** *L. sericata*, thorax, dorsal view. **n** *L. cuprina*, thorax, dorsal view. *ant spir* anterior spiracle, *bas* basicosta, *cox str* coxopleural streak, *gen dil* genal dilation, *III ant* third antennal segment, *in ver* inner vertical seta, *h cal* humeral callus, *kat ter* katatergite, *ntl* notopleuron, *ps g* post gena, *post spir* posterior spiracle, *sub scl* subcostal sclerite

**Fig. 4:**
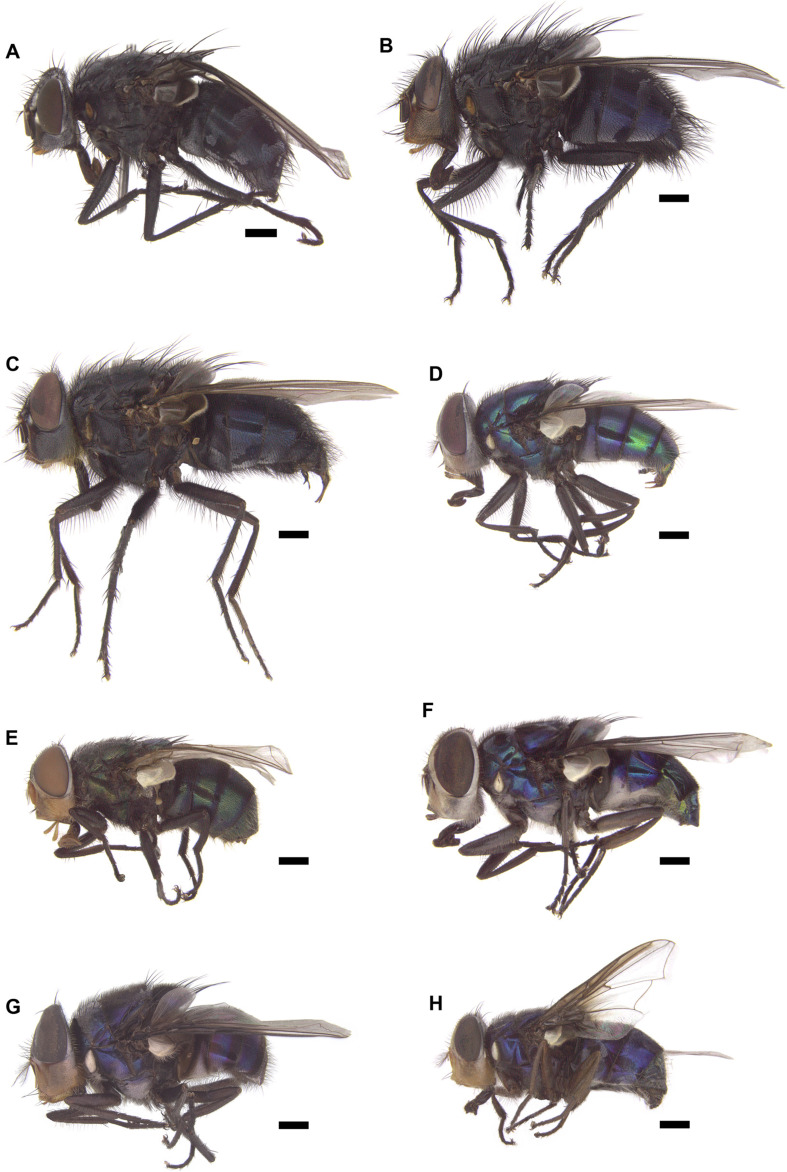
Morphology of African blowflies of forensic importance. **a** *C. croceipalpis.* **b** *C. vicina.* **c** *C. vomitoria.* **d** *Ch. albiceps.* **e** *Ch. bezziana.* **f** *Ch. chloropyga.* **g** *Ch. inclinata.* **h** *Ch. laxifrons*. Scale bar 1 mm

**Fig. 5:**
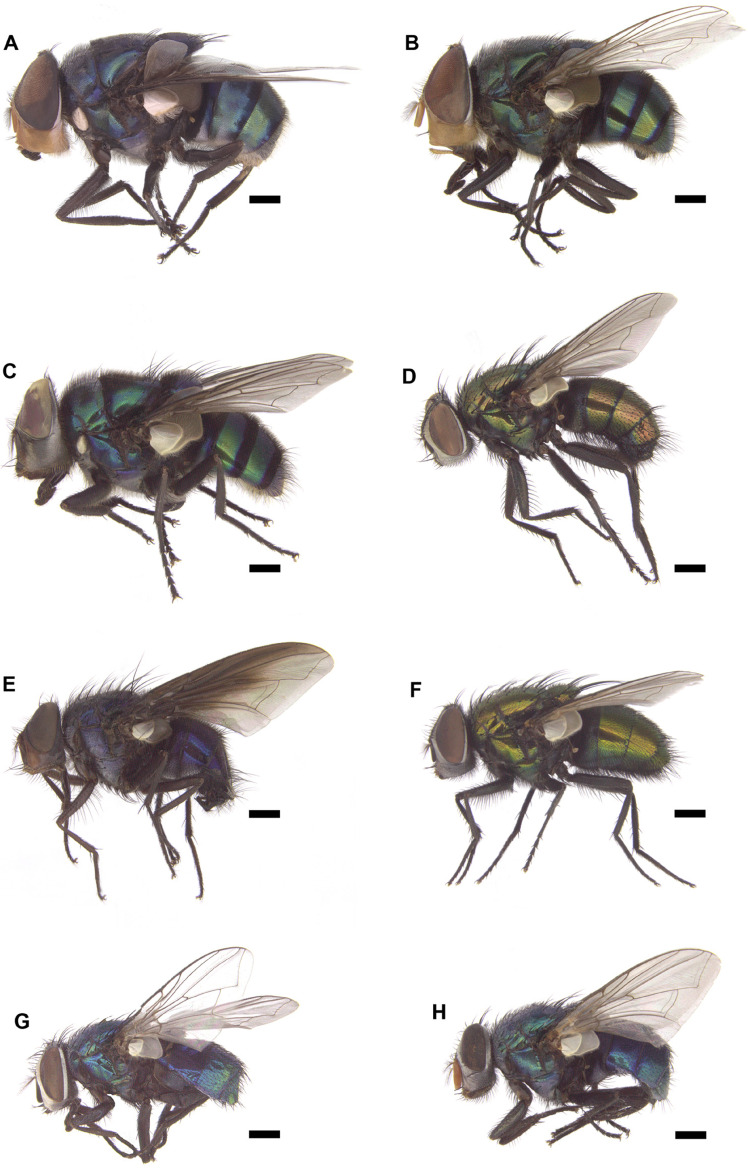
Morphology of African blowflies of forensic importance. **a** *Ch. marginalis*. **b** *Ch. megacephala*. **c** *Ch. putoria*. **d** *L. cuprina*. **e** *L. infernalis*. **f** *L. sericata*. **g** *H. fernandica*. **h** *H. pulchra*. Scale bar 1 mm

1. Stem vein of wing without row of hairs on dorsal surface, greater ampulla without stiff, erect hairs (Fig. 1e) ........................................................................................ 2.

- Stem vein of wing with row of hairs on dorsal surface (Fig. 1a), greater ampulla with stiff, erect hairs (Fig. 1d) ........................................................................................ 3.

2. Lower calypter dark with hairs on dorsal surface (Fig. 1b), thorax non-metallic, dark, and dusted (Figs. 1b, 4a–c); coxopleural streak absent (Fig. 3i); abdomen dark metallic blue, rarely dark green, or olive-green (Fig. 4a–c) ...................................................................................... 10.

- Lower calypter bright white or yellowish, sometimes blackened posteriorly (in *L. infernalis*), always without hairs on dorsal surface (Fig. 1c); thorax metallic green, bluish or coppery (Fig. 5d-h); coxopleural streak present (Fig. 3j); abdomen bright metallic green, blue to coppery (Fig. 5d-h) ...................................................................... 12.

3. Anterior spiracle blackish brown (Figs. 1e, 3b, 4e, 5b) ........................................................................................ 4.

- Anterior spiracle white or yellowish (Figs. 1d, 2h, i, 4f–h, 5a, c) .............................................................................. 5.

4. Eyes of male touching and with sharply demarcated and strikingly large upper facets (Fig. 1f, g); frons of female with fronto-orbital plate dark (Fig. 1h); lower calypter brownish with dark rim (Fig. 5b) .......................................................... *Chrysomya megacephala* (Fabricius, 1794)
  Distribution: Angola, Benin, Botswana, Burkina Faso, Burundi, Cameroon, Central African Republic, Congo, DR Congo, Djibouti, Egypt, Equatorial Guinea, Eritrea, Ethiopia, Gabon, Gambia, Guinea, Ivory Coast, Kenya, Lesotho, Liberia, Libya, Malawi, Mali, Mauritania, Mozambique, Namibia, Niger, Nigeria, Rwanda, Senegal, Sierra Leone, Somalia, South Africa, Sudan, Tanzania, Togo, Uganda, Zambia, Zimbabwe.

- Eyes of male close but not touching and without demarcation between dorsal and ventral facets (Fig. 1i, j); frons of female with fronto-orbital plate reddish (Fig. 1k); lower calypter uniformly white (Fig. 4e) ..................... *Chrysomya bezziana* (Villeneuve, 1914)
  Distribution: Algeria, Cameroon, Chad, Ethiopia, Ivory Coast, Kenya, South Africa, Tanzania, Zambia, Zimbabwe.

5. Anterior wing veins and membrane black or strongly infuscated (Fig. 2e) ........................................................ 6.

- Anterior wing margin hyaline; if weakly darkened (some specimens of *Ch. putoria*), then ground colour of face and genal dilation dark (Fig. 2i) ........................................... 8.

6. Eyes of male with sharply demarcated and strikingly large upper facets (Fig. 2d); fronto-orbital plate of female totally orange (Fig. 2a) ............................................................................ *Chrysomya marginalis* (Wiedemann, 1830) [=*Ch. regalis* Robineau-Desvoidy, 1830]
  Distribution: Burkina Faso, Central African Republic, Egypt, DR Congo, Kenya, Mali, Namibia, Nigeria, Rwanda, Senegal, Somalia, South Africa, Sudan, Tanzania, Zimbabwe.

- Eyes of male without demarcation between dorsal and ventral facets; fronto-orbital plate in female dark at least in upper part (Fig. 2b, c) ................................................ 7.

7. Eyes of male widely separated, frons at its narrowest point measuring 0.5 or more times eye width; frons of female subparallel, fronto-orbital plate with upper part black and lower part bright, reddish (Fig. 2b) ............................................................... *Chrysomya laxifrons* (Villeneuve, 1914)
  Distribution: Cameroon, Central African Republic, DR Congo, Equatorial Guinea, Namibia, Sierra Leone, South Africa, Sudan, Tanzania, Togo, Uganda, Zimbabwe.

- Eyes of male touching (like on Fig. 2d); frons of female conspicuously narrowed on lower half, fronto-orbital plate entirely black (Fig. 2c) ........................................ ................................ *Chrysomya inclinata* (Walker, 1861)
  Distribution: Burkina Faso, Cameroon, Central African Republic, Congo, DR Congo, Eritrea, Ghana, Guinea, Ivory Coast, Liberia, Mozambique, Nigeria, Sierra Leone, South Africa, Tanzania, Togo, Zimbabwe.

8. Dorsal surface of thorax shining green with little, evenly distributed dusting; gena reddish-yellowish in ground colour (Fig. 2h); lower calypter white (Figs. 1d, 4d); outer vertical seta of male well-developed (Fig. 2f) ....................................... *Chrysomya albiceps* (Wiedemann, 1819)
  Distribution: Algeria, Angola, Benin, Botswana, Burkina Faso, Burundi, Cameroon, Central African Republic, Congo, DR Congo, Djibouti, Egypt, Equatorial Guinea, Eritrea, Ethiopia, Gabon, Gambia, Guinea, Ivory Coast, Kenya, Lesotho, Liberia, Libya, Malawi, Mali, Mauritania, Morocco, Mozambique, Namibia, Niger, Nigeria, Rwanda, Senegal, Sierra Leone, Somalia, South Africa, Sudan, Swaziland, Tanzania, Togo, Tunisia, Uganda, Western Sahara, Zambia, Zimbabwe. The most common necrophagous blowfly in Africa.

- Dorsal surface of thorax with conspicuous whitish dusting, forming characteristic patterns of dusted and undusted surfaces (Fig. 2j, k); gena dark in ground colour (Fig. 2i); lower calypter darker (Figs. 4f, 5c); outer vertical seta of male absent (Fig. 2g) .................................... 9.

9. Presutural area of thorax with conspicuous black L-shaped marking on each side of midline, strongly white-dusted median vitta proceeding forwards from suture to front of thorax (Fig. 2j); abdomen with last two segments shiny brassy/green and contrasting strongly with bluish anterior segments (Fig. 2l) ......................................... .................... *Chrysomya chloropyga* (Wiedemann, 1818)
  Distribution: Angola, Benin, Botswana, Burkina Faso, Burundi, Cameroon, Central African Republic, Congo, DR Congo, Djibouti, Equatorial Guinea, Eritrea, Ethiopia, Gabon, Gambia, Guinea, Ivory Coast, Kenya, Lesotho, Liberia, Malawi, Mali, Mauritania, Mozambique, Namibia, Niger, Nigeria, Rwanda, Senegal, Sierra Leone, Somalia, South Africa, Sudan, Tanzania, Togo, Uganda, Zambia, Zimbabwe.

- Presutural area of thorax without conspicuous paired L-shaped markings, but with vague lateral dark spots, white-dusted median vitta extending forwards hardly halfway to front of thorax (Fig. 2k); abdomen with last two segments concolourous with green/bluish anterior segments (Fig. 2m) ................................................................... *Chrysomya putoria* (Wiedemann, 1830)
  Distribution: Benin, Botswana, Burkina Faso, Cameroon, Central African Republic, DR Congo, Gambia, Ghana, Guinea, Eritrea, Ethiopia, Ivory Coast, Kenya, Malawi, Mali, Mozambique, Namibia, Senegal, South Africa, Sudan, Swaziland, Tanzania, Uganda, Zambia, Zimbabwe.

10. Anterior part of genal dilation yellow to orange (Fig. 3a); basicosta bright to dark yellow ........................................................ *Calliphora vicina* (Robineau-Desvoidy, 1830) [=*C. erythrocephala* (Meigen, 1826)]
  Distribution: Algeria, Egypt, Morocco, South Africa. Possibly occur in all countries of North Africa.

- Anterior part of genal dilation uniformly black (Fig. 3b, c); basicosta brownish-black ........................................11.

11. Hairs on genal dilation and postgena orange (Fig. 3b), anterior spiracle brownish-black (Fig. 3b) ................... *Calliphora vomitoria* (Linnaeus, 1758)
  Distribution: Morocco. Erzinçlioğlu (1987) gives general information “North Africa”. Probably distributed in mountainous areas of North Africa.

- Hairs on genal dilation and postgena predominantly black (Fig. 3c); anterior spiracle dark yellow (Fig. 3c) .......................... *Calliphora croceipalpis* Jaennicke, 1867
  Distribution: DR Congo, Eritrea, Ethiopia, Malawi, Rwanda, South Africa, Tanzania, Zimbabwe. Widespread from East to South Africa [6].

12. Katatergite (small pleurite on lateral surface of thorax, situated above posterior spiracle, see Fig. 1b) bare or pubescent, without long, erect hairs ............................13.

- Katatergite with long, fine, erect hairs (Fig. 3d) ...........15.

13. Wing deeply infuscated along anterior margin (Figs. 2e, 5e); subcostal sclerite with one or more black setulae (Fig. 3e) ................. *Lucilia infernalis* Villeneuve, 1914
  Distribution: Burundi, Cameroon, Congo, DR Congo, Equatorial Guinea, Ghana, Liberia, Malawi, Nigeria, Rwanda, Uganda, Tanzania, Togo, Zimbabwe. Rare species but probably distributed over the whole rain forest area of Africa [23].

- Wing transparent (Fig. 5d, f); hairs on subcostal sclerite only pubescent (Fig. 3f) .............................................. 14.

14. Metasternal area (sclerite midventrally between middle and hind coxae) setose; posterior slope of humeral callus with 6–8 hairs (Fig. 3m); surface of notopleuron between last notopleural seta and edge of notopleuron with 8–16 hairs (Fig. 3m); central occipital area with 2–8 setulae below each inner vertical seta (Fig. 3g) .................................................................. *Lucilia sericata* (Meigen, 1826)
  Distribution: Algeria, Egypt, Lesotho, Libya, Mozambique, Namibia, South Africa, Zimbabwe. Possible occurrence in all countries of the North Africa.

- Metasternal area bare; posterior slope of humeral callus with 0–4 hairs (Fig. 3n); surface of notopleuron between last notopleural seta and edge of notopleuron with only 2–5 hairs (Fig. 3n); central occipital area with one setula (rarely 0 or 2) below each inner vertical seta (Fig. 3h) ....................................................... *Lucilia cuprina* (Wiedemann, 1830)
  Distribution: Botswana, Cameroon, DR Congo, Ethiopia, Kenya, Lesotho, Liberia, Malawi, Mozambique, Namibia, Rwanda, Senegal, Somalia, South Africa, Tanzania, Togo, Uganda, Zimbabwe. Widespread in the Afrotropical Region [24].

15. Third antennal segment bright orange (Fig. 3l); eyes in male almost touching ............................................................................ *Hemipyrellia pulchra* (Wiedemann, 1830)
  Distribution: Egypt, Mozambique, Nigeria, Sudan. Widespread West to East Africa [6].

- Third antennal segment dark (Fig. 3k); in male head sub-holoptic with eyes broadly separated (Fig. 3k) ..................... *Hemipyrellia fernandica* (Macquart, 1855)
  Distribution: Benin, Botswana, Burundi, Cameroon, Central African Republic, Democratic Republic of Congo, Gabon, Gambia, Ghana, Ivory Coast, Liberia, Malawi, Mozambique, Namibia, Nigeria,? Senegal, South Africa, Sudan, Tanzania, Togo, Zimbabwe. Widespread in the Afrotropical Region [24].

## Discussion

The list of species of forensic importance was prepared for regions with well-studied faunas, usually based on information from human forensic cases or ecological succession studies of large-bodied carrion [1, 37, 38]. Szpila and Villet [39] proposed a list of ten common necrophagous blowflies which may be found on human corpses across the continent, and which they stated should be extended to include additional widespread species. The present paper expands this list by five other necrophagous species (*Ch. inclinata*, *Ch. laxifrons*, *L. infernalis*, *H. pulchra*, *H. fernandica*) and one obligate parasite of vertebrates (*Ch. bezziana*) that might affect some forensic cases.^1^^*^

Studies on regional African necrophagous faunas are scarce [1, 38] and mostly concern South Africa [40–48], Egypt [49–52], and Nigeria [35, 53, 54], with a few from Zimbabwe [55], Cameroon [56], and Algeria [36]. Of course, the African blowfly fauna still needs more faunistic studies, but the presented key should be successfully applicable in most continental African countries, especially in anthropogenic habitats.

The morphology and taxonomy of adult forms of common necrophagous blowflies is relatively well known, both worldwide and for African species [1, 3, 23–25, 30, 32]. However, consolidation of these data into one key revealed a few problems in designing couplets for some closely related species. The most familiar is the identification of *L. cuprina* and *L. sericata* and their possible hybrids, which has fortunately been deeply studied [32, 57–59], especially for African populations. As a result, a relatively broad set of morphological characters for the separation of both species was presented [32].

*Calliphora croceipalpis* and *C. vicina* have never been put together in one key dedicated to adult forms [3, 23, 24]. The latter species is considered introduced and invasive in South Africa [1, 6, 60] and, as a relatively new element of the Afrotropical fauna, was not included in Zumpt’s regional taxonomic monograph [23], although both are discussed in his myiasis monograph [3].

The darkened anterior wing margin in *Chrysomya* may also be confusing. In *Ch. inclinata*, *Ch. laxifrons*, and *Ch. marginalis*, the black veins and membrane of the margin of mature adults are obvious, easily observed, and quickly separate these species from other members of the genus, which lack pigment or, in some specimens of *Ch. putoria* (‘f. *taeniata* Bigot’ sensu Zumpt [3, 23]), have more-or-less darkened (but not black) veins. Both immature and partially teneral adults of the three species of the black-edged group may be only darkened, and in this case, distinction is made by checking the ground colour of the face and genal dilation (bright yellowish-orange in *Ch. inclinata*, *Ch. laxifrons*, and *Ch. marginalis*, and dark in *Ch. putoria*). It is important to mention that two rare East African montane species with a darkened anterior wing margin are not included in the present key: it is likely but not yet certain that *Ch. polymita* (Villeneuve 1914) and *Ch. vanemdeni* (Zumpt 1953) breed in carrion [23]; both possess a large, dark glossy spot on the genal dilation [25, 30]. They are characterised by Zumpt [23] and illustrated by Irish et al. [25].

Published information about the distribution of individual species of necrophagous blowflies in particular African countries is often confusing. Most of these species are widespread on the continent, and the Catalogue of the Diptera of the Afrotropical Region [6] provided data that are often restricted to comments like e.g., “widespread E. Afr. to sthn Afr.” (*C. croceipalpis*), “… widespread Afrotrop. Reg.” (*H. fernandica*), or “… widespread mainland Afrotropical Region” (*L. infernalis*). On the other hand, Verves [34] published very detailed lists of African countries for distribution of five species (*Ch. albiceps*, *Ch. chloropyga*, *Ch. megacephala*, *L. infernalis*, and *H. fernandica*) without any mention of the source of this information (and before Rognes and Paterson clarified the independence of *Ch. chloropyga* and *Ch. putoria* [30]). Additionally, in Irish et al. [25], information about the distribution of *Ch. albiceps*, *Ch. chloropyga*, and *Ch. marginalis* is mismatched between the text and maps. Contrary to the text information, Madagascar and Egypt are omitted on the maps for *Ch. albiceps* and *Ch. marginalis*, respectively, and Egypt is additionally marked on the map for *Ch. chloropyga*. Fortunately, these cases are easy to clarify, and it is clear that the distribution description in the text is congruent with the existing distributional data [6, 34]. Generally, these problems indicate a strong need for studies of local fauna profiled toward species of forensic importance in most African countries.

This key will assist entomologists on the African continent to use flies in forensic cases and push forward the use of forensic entomology in Africa, as well as the fields of public health and human and veterinary medicine. Further taxonomic research should focus on the alternative morphological methods of identification of adult flies [61, 62] and identification of preimaginal instars, as it is systematically implemented on other continents [63, 64]. This will facilitate large-scale research on spatial and seasonal distribution of forensically important flies in different African countries that is crucial for future forensic work.

## Electronic supplementary material

ESM 1 (PDF 865 kb)

## Footnotes

1
^*^Forensic cases in Africa and Asia include murders, neglect, and poaching. Adult screwworms that visit wounds, if only to feed, may be collected by death investigators. Cases of criminal neglect and poaching are not always immediately lethal, and adult screwworms might be recovered from cases involving live or dead humans and other animals.
