# Supplementary material for: Species identification of adult African blowflies (Diptera: Calliphoridae) of forensic importance
Source: Int J Legal Med. 2017 Aug 28;132(3):831–42. doi: 10.1007/s00414-017-1654-y (PMC5919996; doi:10.1007/s00414-017-1654-y)
Supplement: Supplementary file 1 [file 414_2017_1654_MOESM1_ESM.pdf]

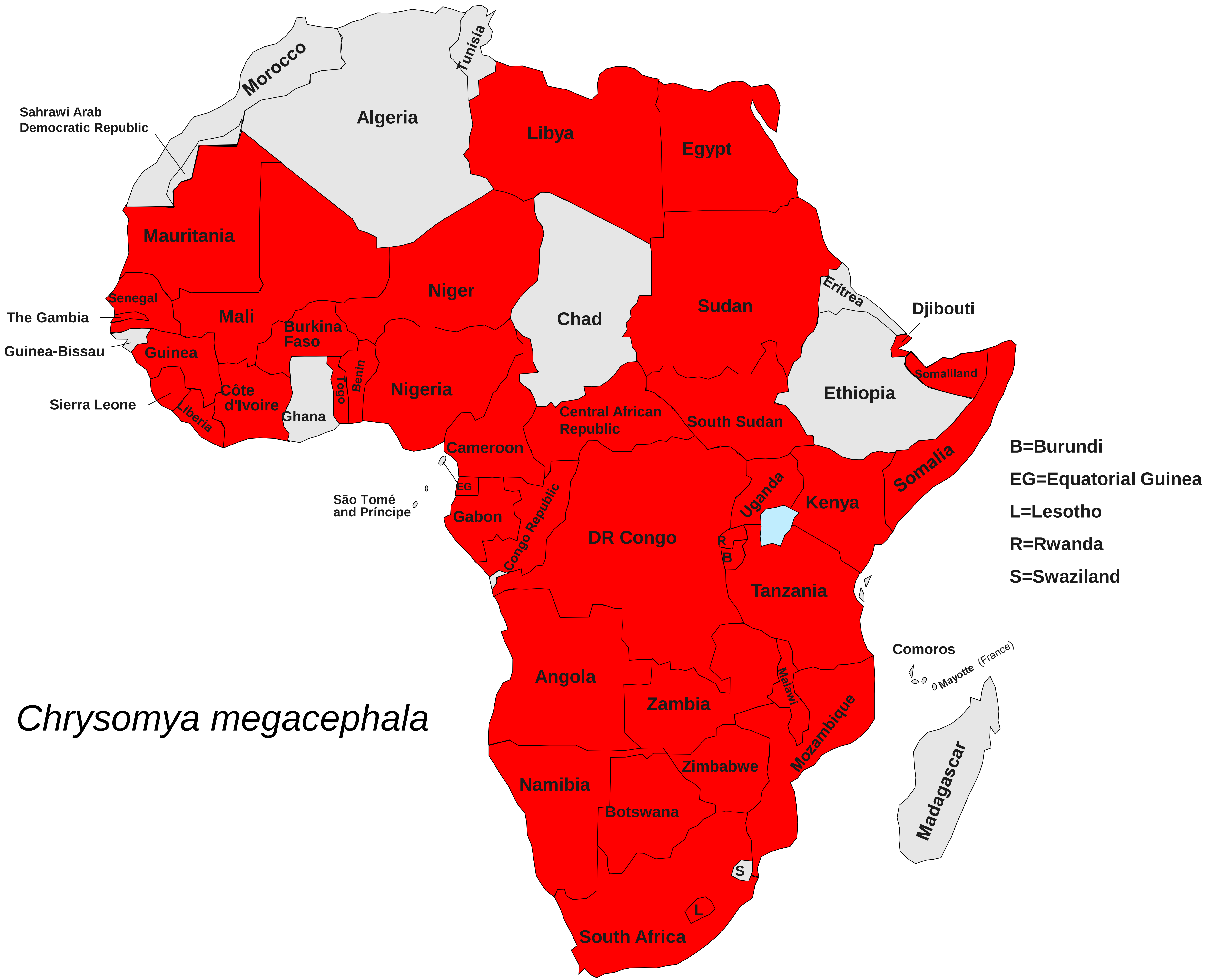
Morocco
Tunisia
Algeria
Libya
Egypt
Sahrawi Arab
Democratic Republic
Mauritania
Senegal
The Gambia
Guinea-Bissau
Guinea
Sierra Leone
Liberia
Mali
Burkina
Faso
Côte
d'Ivoire
Ghana
Togo
Benin
Niger
Nigeria
Chad
Sudan
Eritrea
Djibouti
Ethiopia
Somaliland
Somalia
Central African
Republic
South Sudan
Cameroon
EG
São Tomé
and Príncipe
Gabon
Congo Republic
DR Congo
Uganda
Kenya
R
B
Tanzania
Angola
Zambia
Malawi
Mozambique
Comoros
Mayotte
(France)
Madagascar
Zimbabwe
Namibia
Botswana
S
L
South Africa
B=Burundi
EG=Equatorial Guinea
L=Lesotho
R=Rwanda
S=Swaziland
Chrysomya megacephala

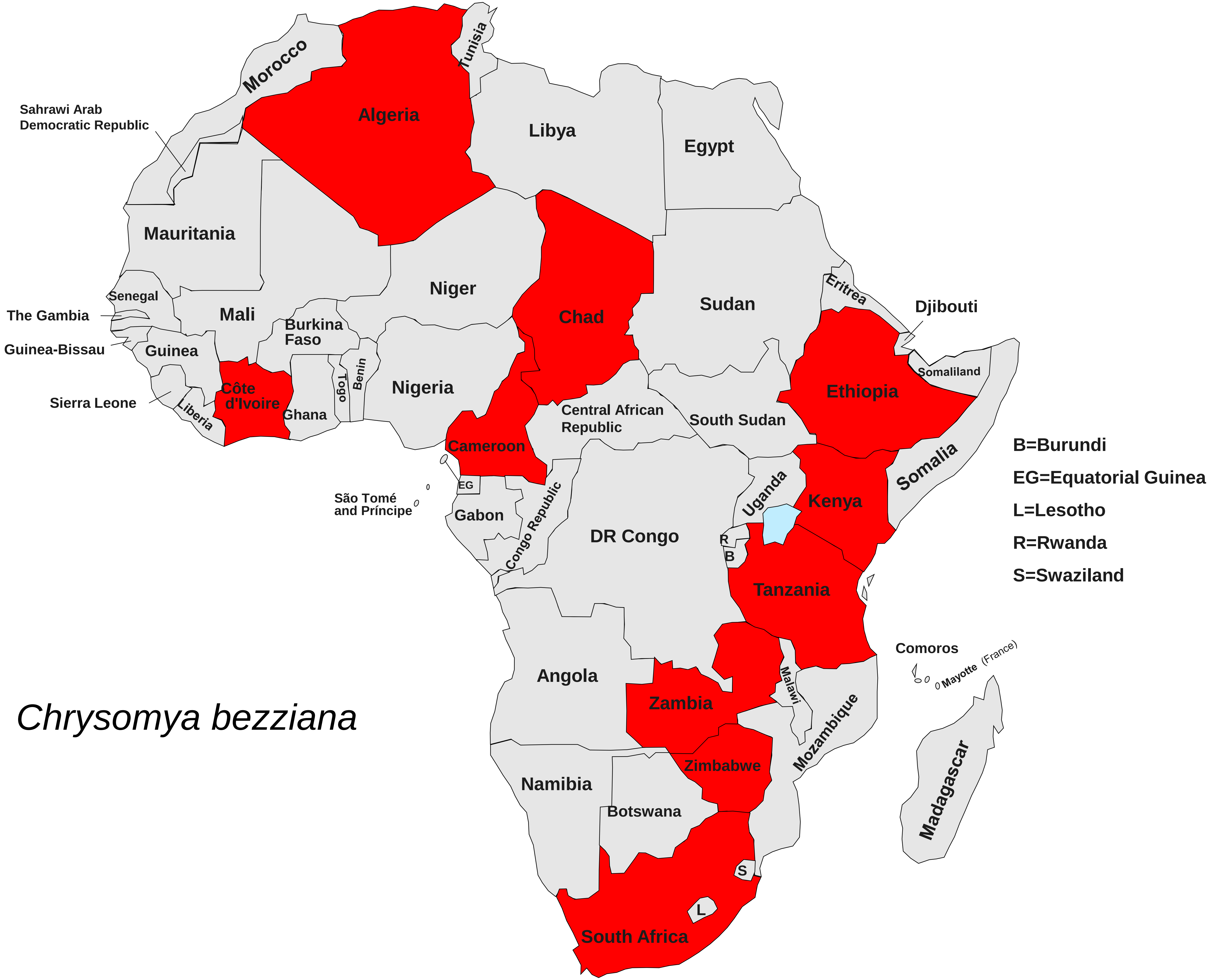

Chrysomya bezziana

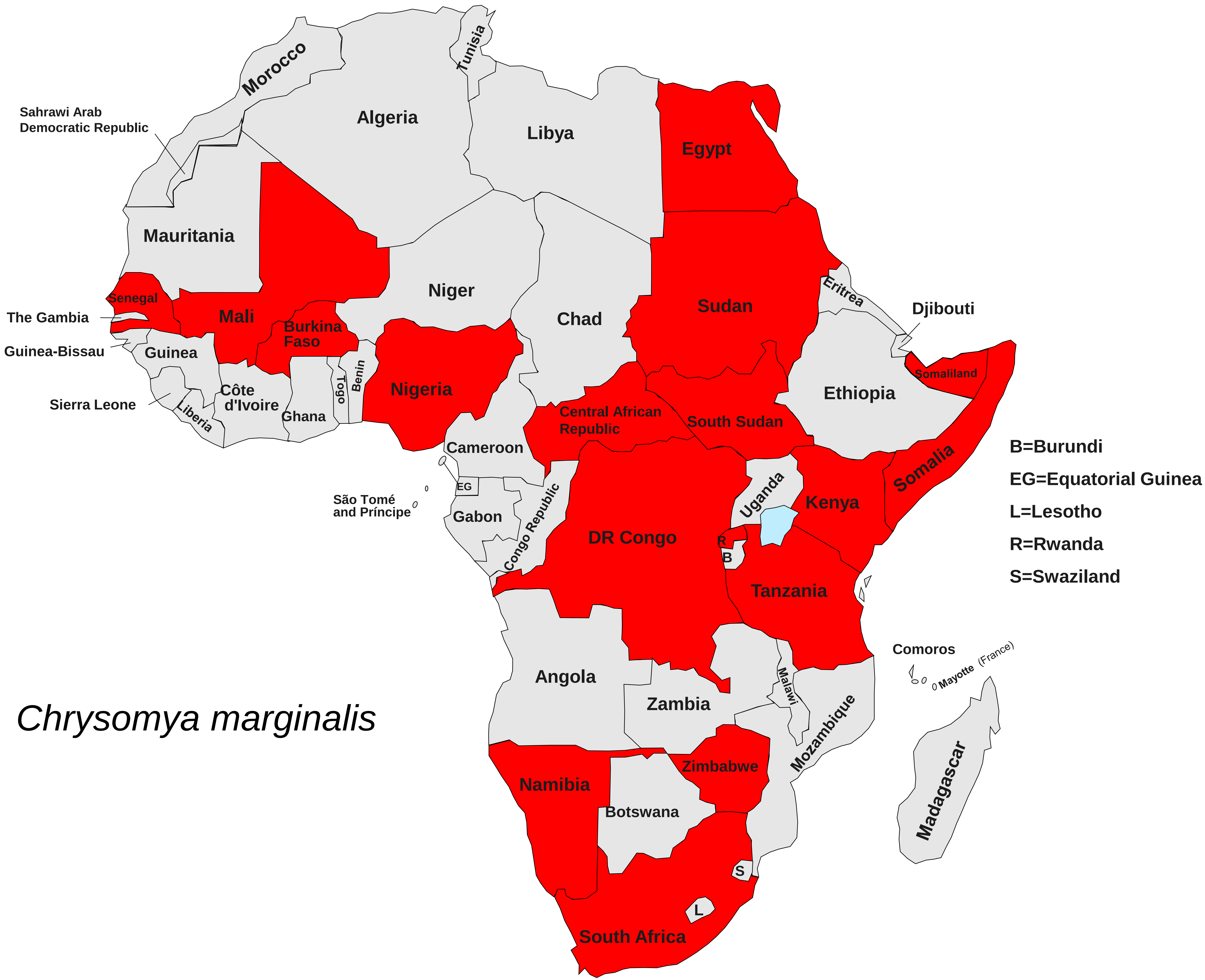
Chrysomya marginalis
Morocco
Tunisia
Algeria
Libya
Egypt
Sahrawi Arab Democratic Republic
Mauritania
Mali
Niger
Chad
Sudan
Eritrea
Djibouti
Senegal
The Gambia
Guinea-Bissau
Guinea
Burkina Faso
Sierra Leone
Liberia
Côte d'Ivoire
Ghana
Togo
Benin
Nigeria
Central African Republic
South Sudan
Ethiopia
Somaliland
Somalia
Cameroon
EG
São Tomé and Príncipe
Gabon
Congo Republic
DR Congo
Uganda
Kenya
R
B
Tanzania
Angola
Zambia
Malawi
Mozambique
Comoros
Mayotte (France)
Madagascar
Zimbabwe
Namibia
Botswana
S
L
South Africa
B=Burundi
EG=Equatorial Guinea
L=Lesotho
R=Rwanda
S=Swaziland

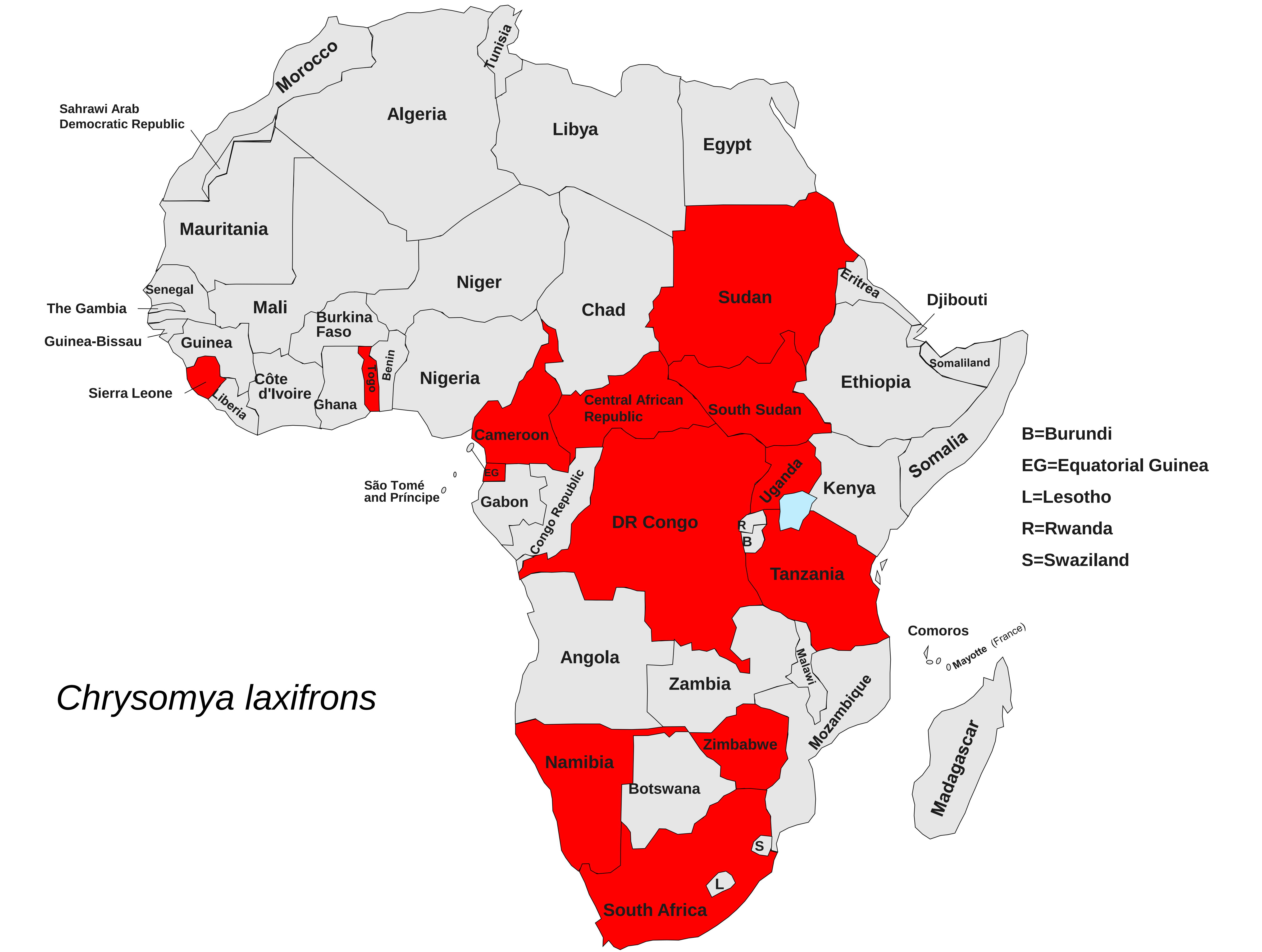
Chrysomya laxifrons
Morocco
Tunisia
Algeria
Libya
Egypt
Sahrawi Arab
Democratic Republic
Mauritania
Mali
Niger
Chad
Sudan
Eritrea
Djibouti
Senegal
The Gambia
Guinea-Bissau
Guinea
Sierra Leone
Liberia
Côte
d'Ivoire
Burkina
Faso
Ghana
Togo
Benin
Nigeria
Cameroon
Central African
Republic
South Sudan
Ethiopia
Somaliland
Somalia
Kenya
Uganda
EG
São Tomé
and Príncipe
Gabon
Congo Republic
DR Congo
R
B
Tanzania
Angola
Zambia
Malawi
Mozambique
Comoros
Mayotte (France)
Madagascar
Namibia
Botswana
Zimbabwe
S
L
South Africa
B=Burundi
EG=Equatorial Guinea
L=Lesotho
R=Rwanda
S=Swaziland

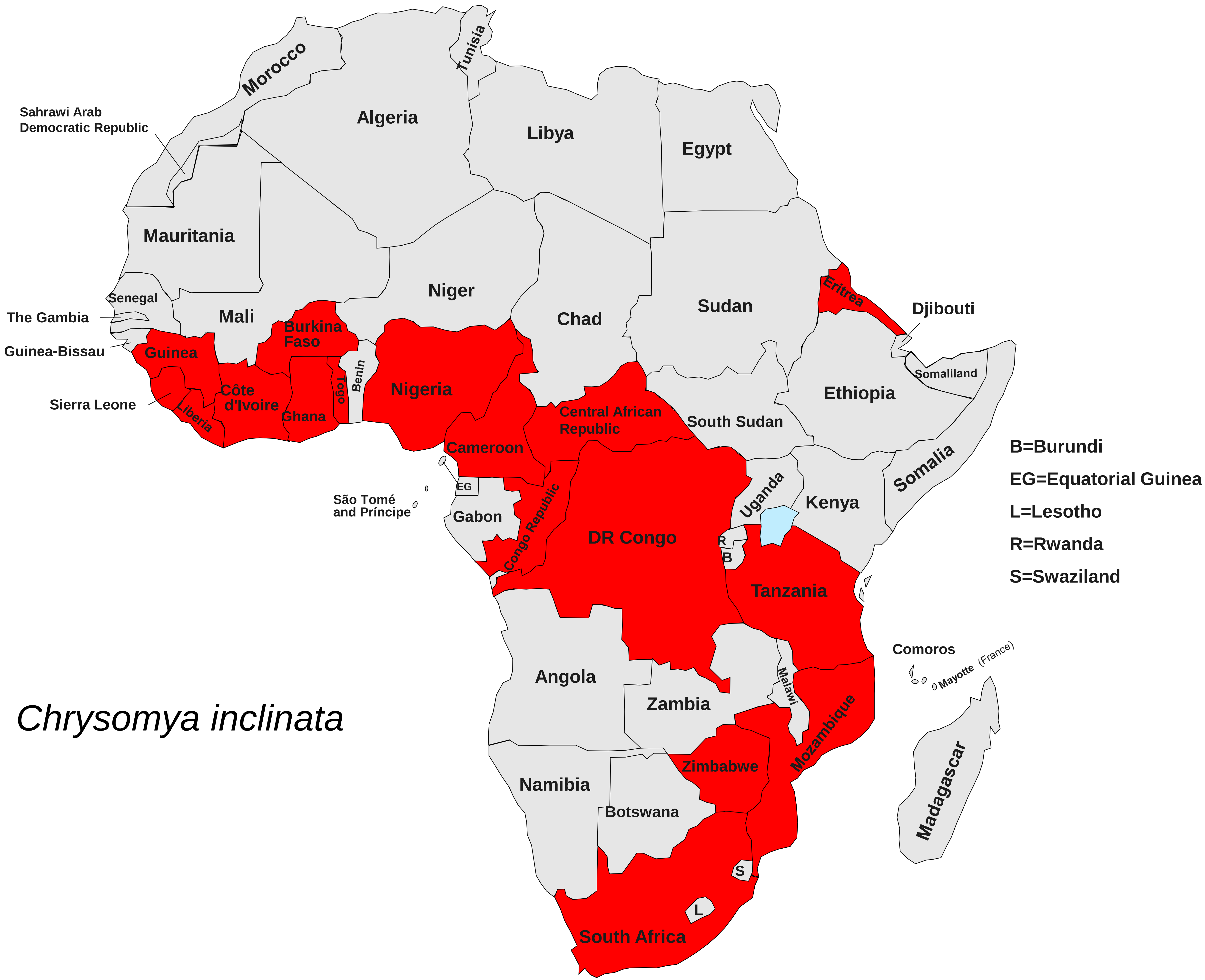
Chrysomya inclinata
Morocco
Tunisia
Algeria
Libya
Egypt
Sahrawi Arab
Democratic Republic
Mauritania
Mali
Niger
Chad
Sudan
Eritrea
Djibouti
Senegal
The Gambia
Guinea-Bissau
Guinea
Burkina
Faso
Sierra Leone
Liberia
Côte
d'Ivoire
Ghana
Togo
Benin
Nigeria
Central African
Republic
South Sudan
Ethiopia
Somaliland
Somalia
Cameroon
EG
São Tomé
and Príncipe
Gabon
Congo Republic
DR Congo
Uganda
Kenya
R
B
Tanzania
Angola
Zambia
Malawi
Mozambique
Comoros
Mayotte (France)
Madagascar
Zimbabwe
Namibia
Botswana
S
L
South Africa
B=Burundi
EG=Equatorial Guinea
L=Lesotho
R=Rwanda
S=Swaziland

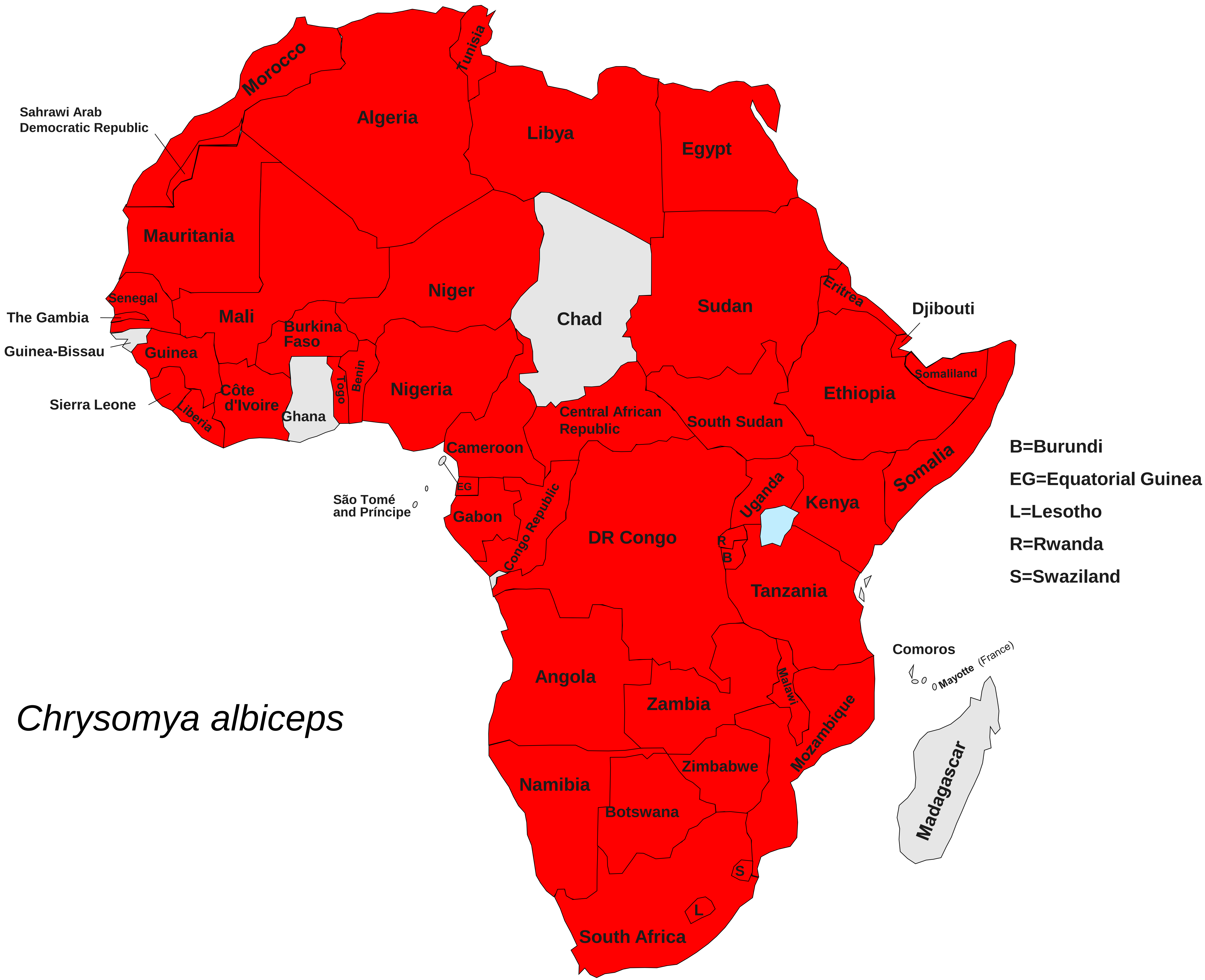
Morocco
Tunisia
Sahrawi Arab
Democratic Republic
Algeria
Libya
Egypt
Mauritania
Niger
Senegal
The Gambia
Mali
Burkina
Faso
Chad
Sudan
Eritrea
Djibouti
Guinea-Bissau
Guinea
Benin
Togo
Nigeria
Ethiopia
Somaliland
Sierra Leone
Côte
d'Ivoire
Liberia
Ghana
Central African
Republic
South Sudan
Cameroon
Somalia
B=Burundi
EG=Equatorial Guinea
L=Lesotho
R=Rwanda
S=Swaziland
EG
Uganda
Kenya
São Tomé
and Príncipe
Gabon
Congo Republic
DR Congo
R
B
Tanzania
Comoros
Mayotte
(France)
Angola
Malawi
Zambia
Mozambique
Madagascar
Chrysomya albiceps
Zimbabwe
Namibia
Botswana
S
L
South Africa

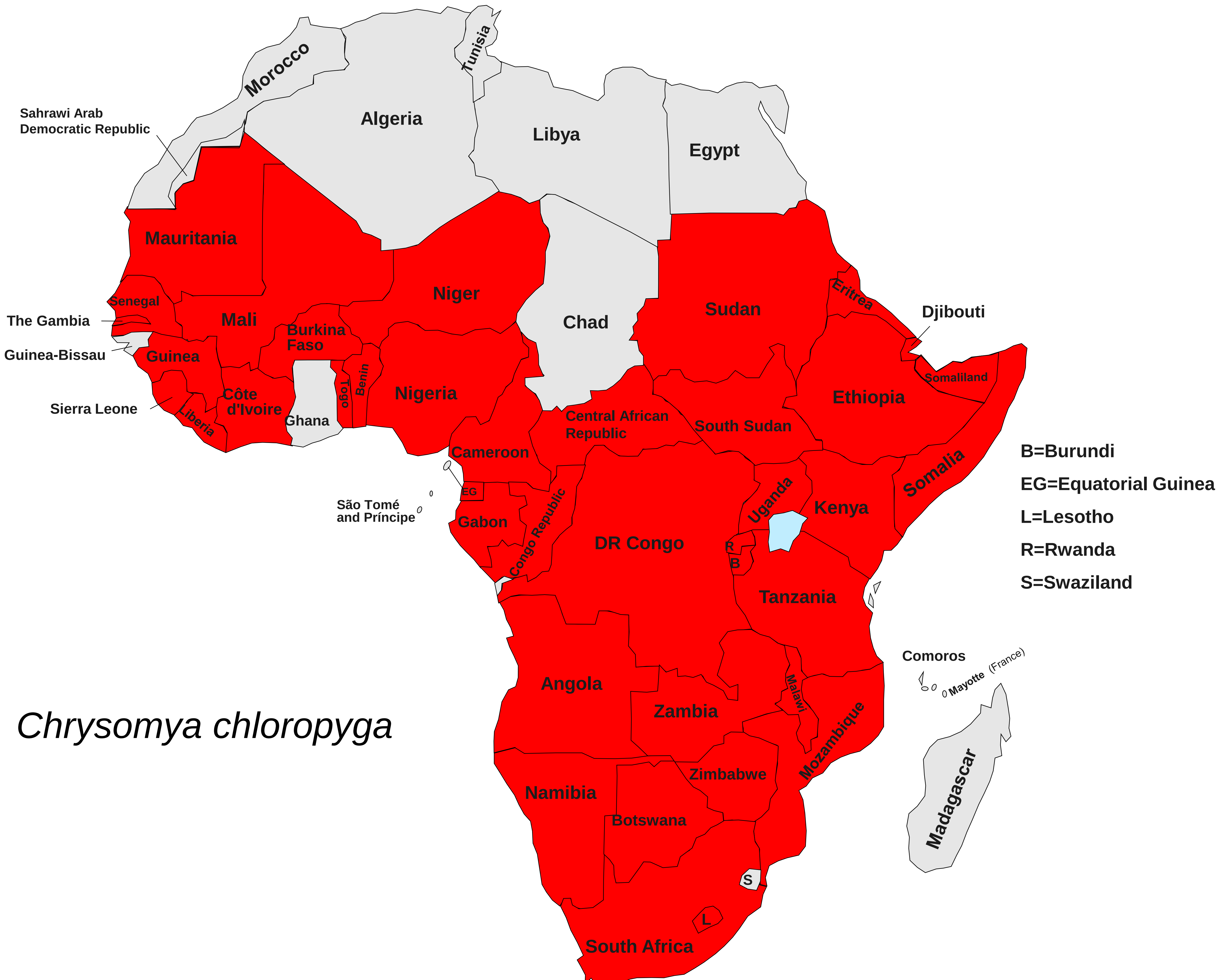
Morocco
Tunisia
Algeria
Libya
Egypt
Sahrawi Arab
Democratic Republic
Mauritania
Senegal
The Gambia
Guinea-Bissau
Guinea
Sierra Leone
Liberia
Mali
Burkina
Faso
Côte
d'Ivoire
Ghana
Togo
Benin
Niger
Nigeria
Chad
Sudan
Eritrea
Djibouti
Somaliland
Ethiopia
South Sudan
Central African
Republic
Cameroon
EG
São Tomé
and Príncipe
Gabon
Congo Republic
DR Congo
Uganda
Kenya
Somalia
R
B
Tanzania
B=Burundi
EG=Equatorial Guinea
L=Lesotho
R=Rwanda
S=Swaziland
Comoros
Mayotte
(France)
Angola
Zambia
Malawi
Mozambique
Madagascar
Zimbabwe
Namibia
Botswana
S
L
South Africa
Chrysomya chloropyga

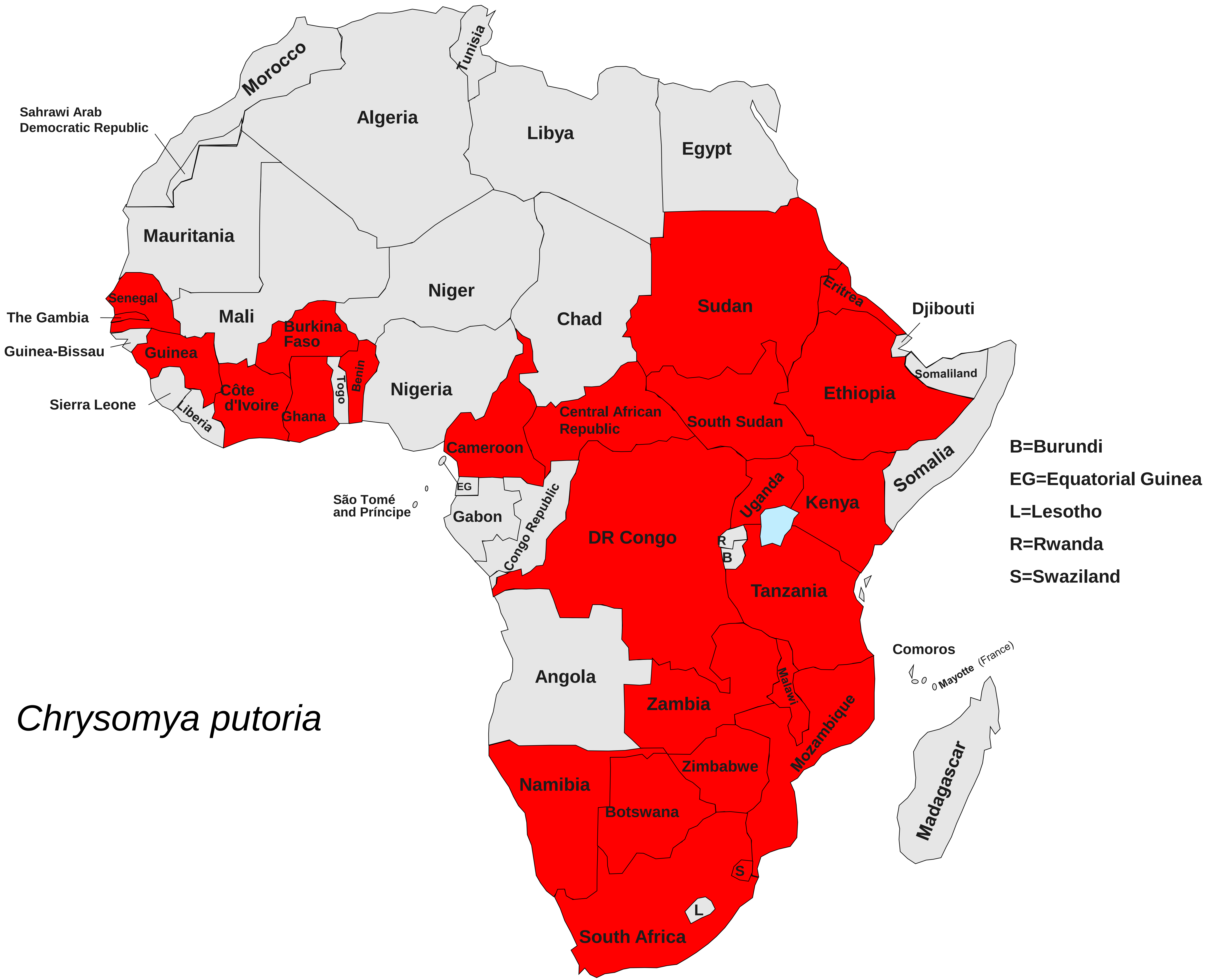
Chrysomya putoria
Morocco
Tunisia
Algeria
Libya
Egypt
Sahrawi Arab
Democratic Republic
Mauritania
Mali
Niger
Chad
Sudan
Eritrea
Djibouti
Senegal
The Gambia
Guinea-Bissau
Guinea
Burkina
Faso
Benin
Togo
Sierra Leone
Liberia
Côte
d'Ivoire
Ghana
Nigeria
Central African
Republic
South Sudan
Ethiopia
Somaliland
Somalia
Cameroon
EG
São Tomé
and Príncipe
Gabon
Congo Republic
DR Congo
Uganda
Kenya
R
B
Tanzania
Angola
Zambia
Malawi
Mozambique
Comoros
Mayotte
(France)
Madagascar
Zimbabwe
Namibia
Botswana
S
L
South Africa
B=Burundi
EG=Equatorial Guinea
L=Lesotho
R=Rwanda
S=Swaziland

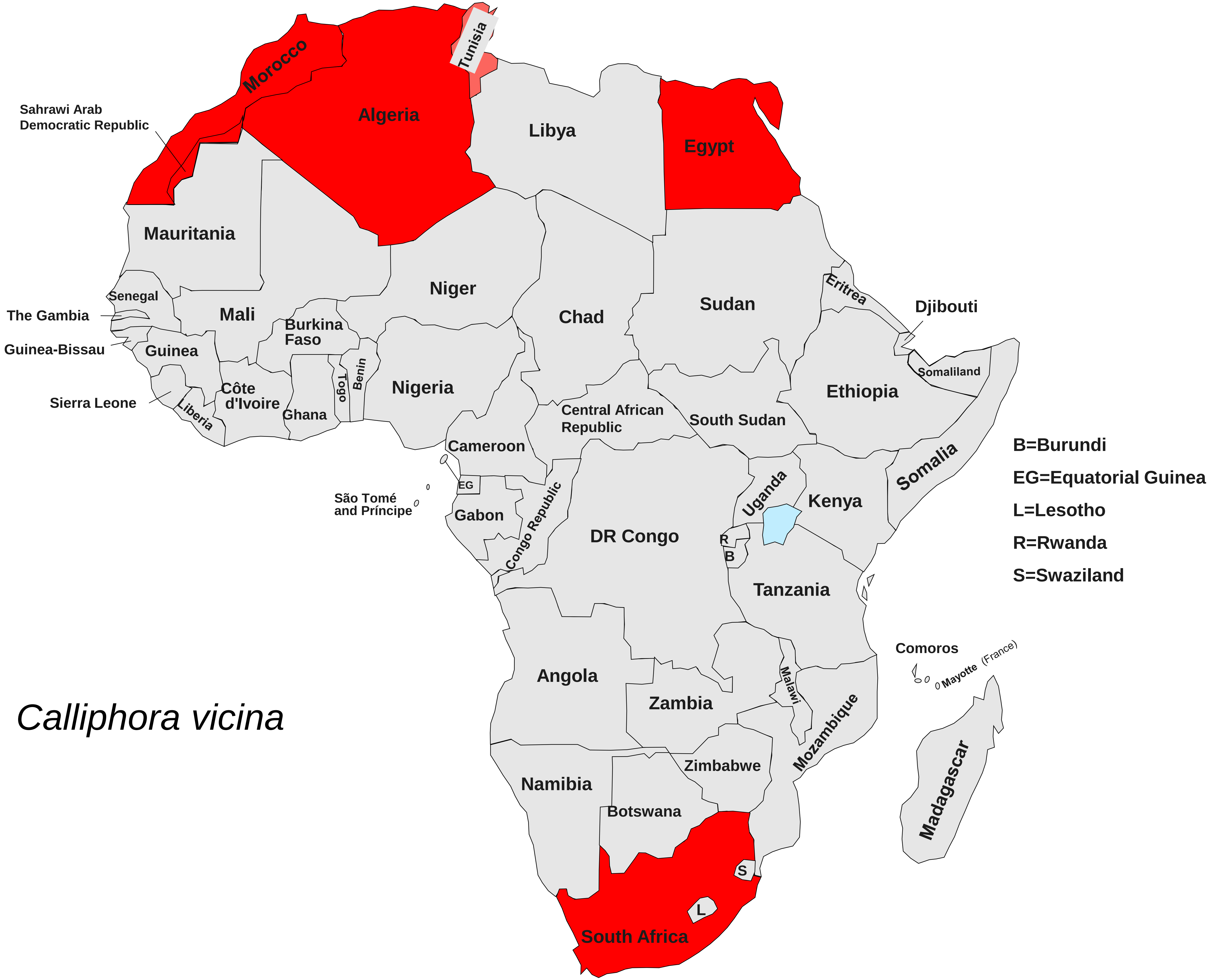

Calliphora vicina

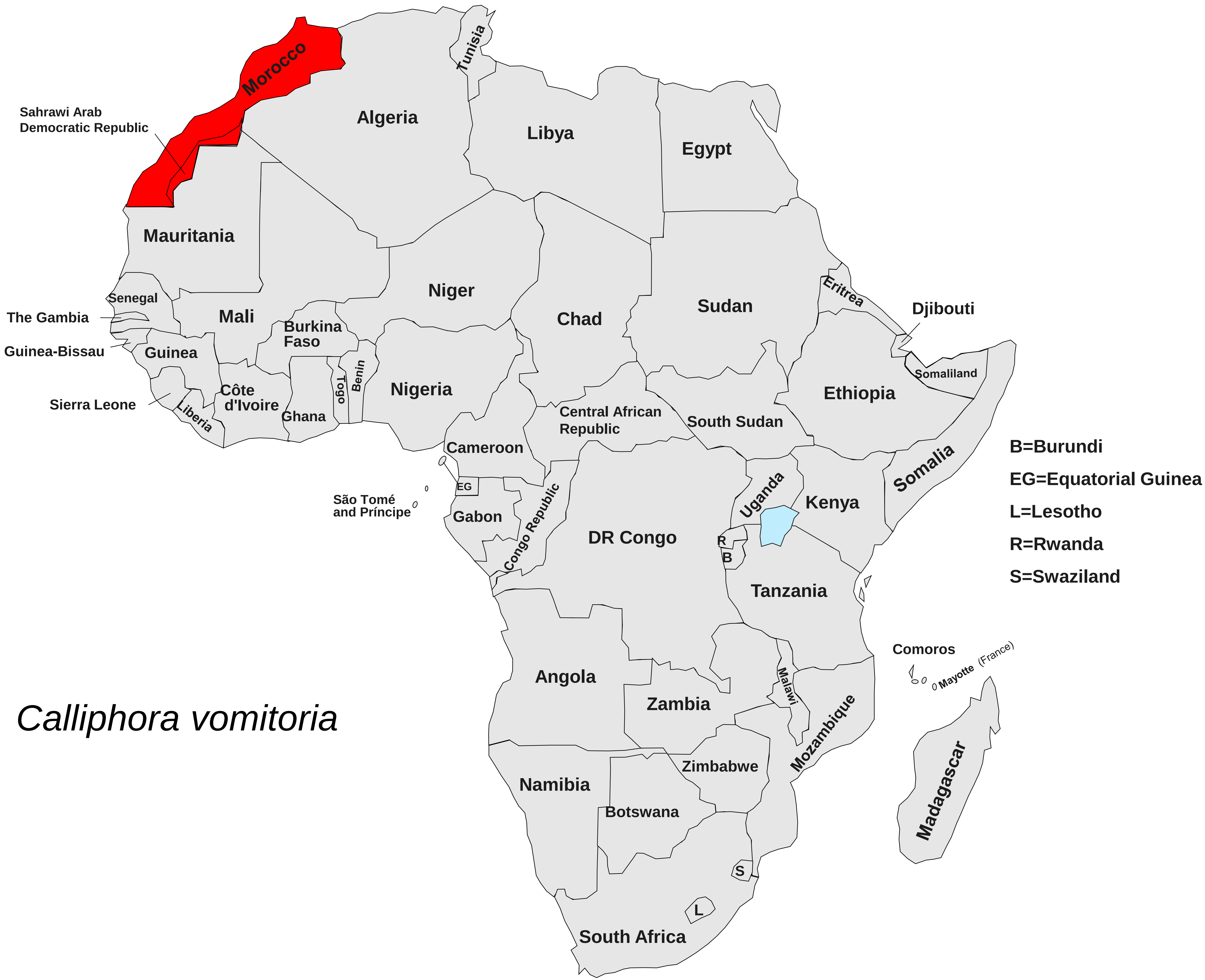

Calliphora vomitoria

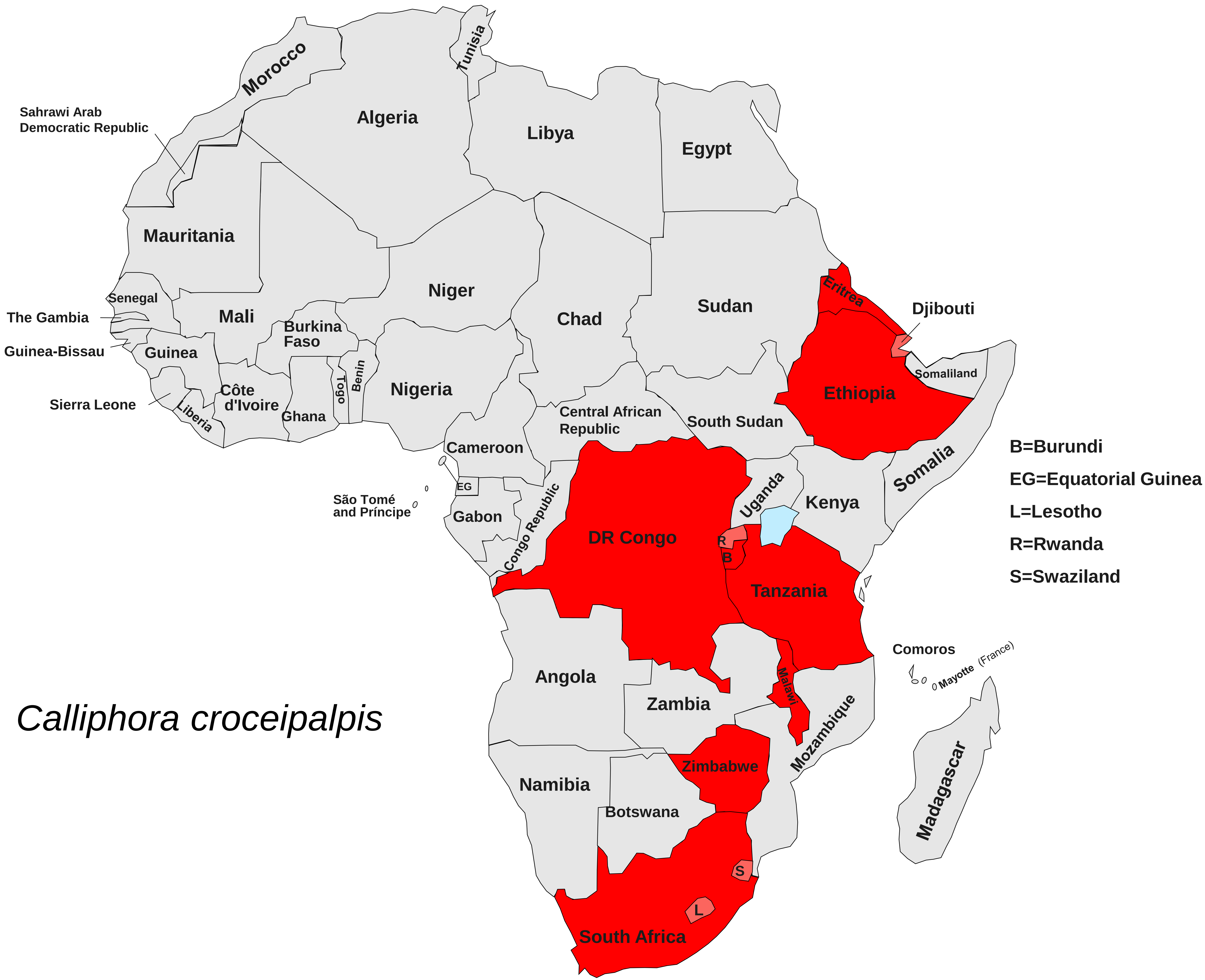

Calliphora croceipalpis
Morocco
Tunisia
Algeria
Libya
Egypt
Sahrawi Arab Democratic Republic
Mauritania
Mali
Niger
Chad
Sudan
Eritrea
Djibouti
Senegal
The Gambia
Guinea-Bissau
Guinea
Sierra Leone
Liberia
Côte d'Ivoire
Burkina Faso
Ghana
Togo
Benin
Nigeria
Central African Republic
South Sudan
Ethiopia
Somaliland
Somalia
Cameroon
EG
São Tomé and Príncipe
Gabon
Congo Republic
DR Congo
Uganda
Kenya
R
B
Tanzania
Angola
Zambia
Malawi
Mozambique
Comoros
Mayotte (France)
Madagascar
Zimbabwe
Namibia
Botswana
S
L
South Africa
B=Burundi
EG=Equatorial Guinea
L=Lesotho
R=Rwanda
S=Swaziland

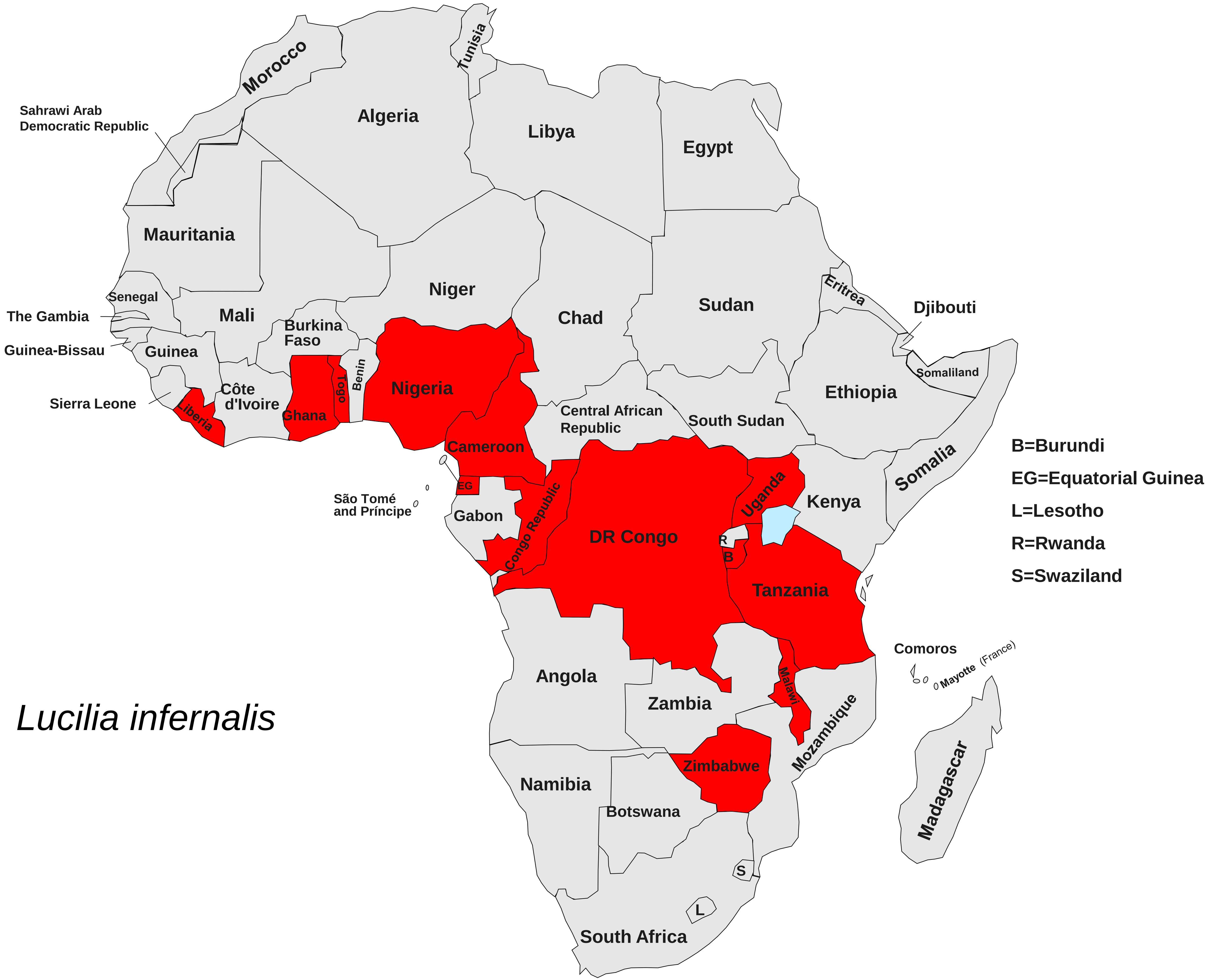

Lucilia infernalis

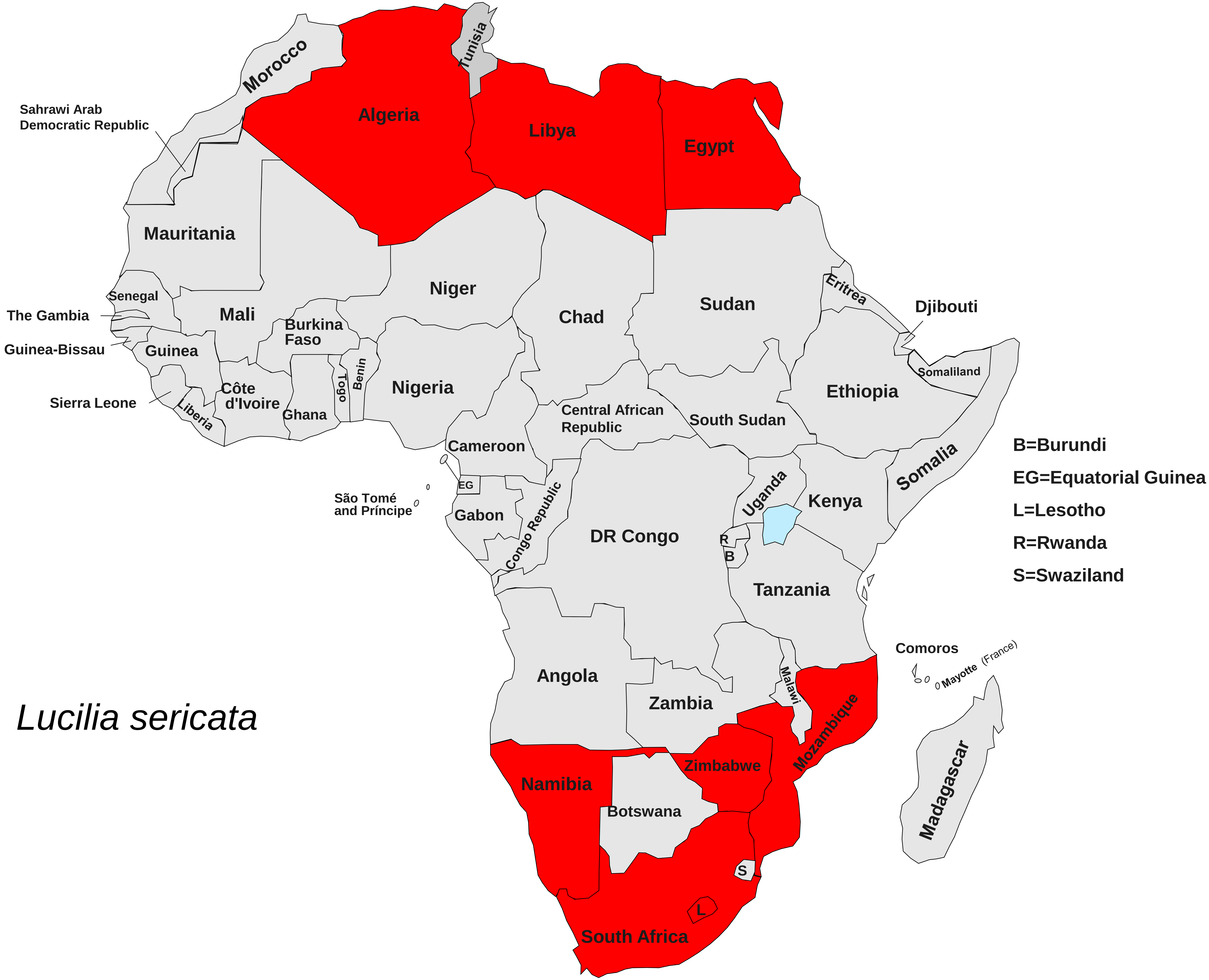

Lucilia sericata

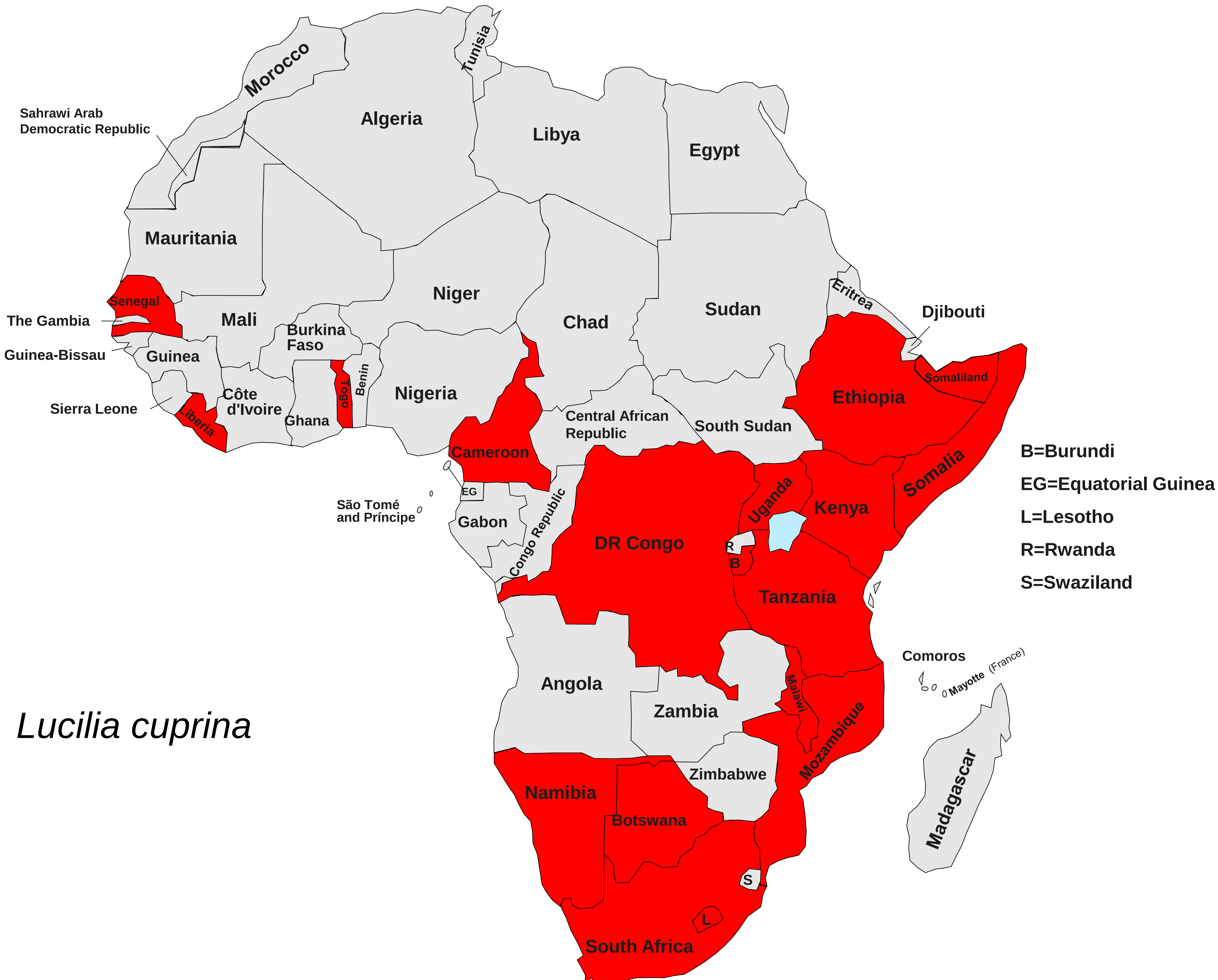
Morocco
Tunisia
Algeria
Libya
Egypt
Sahrawi Arab
Democratic Republic
Mauritania
Mali
Niger
Chad
Sudan
Eritrea
Djibouti
Senegal
The Gambia
Guinea-Bissau
Guinea
Sierra Leone
Liberia
Côte d'Ivoire
Burkina Faso
Ghana
Togo
Benin
Nigeria
Cameroon
Central African Republic
South Sudan
Ethiopia
Somaliland
Somalia
Kenya
Uganda
EG
São Tomé and Príncipe
Gabon
Congo Republic
DR Congo
R
B
Tanzania
Angola
Zambia
Malawi
Mozambique
Comoros
Mayotte (France)
Madagascar
Zimbabwe
Namibia
Botswana
S
L
South Africa
B=Burundi
EG=Equatorial Guinea
L=Lesotho
R=Rwanda
S=Swaziland
Lucilia cuprina

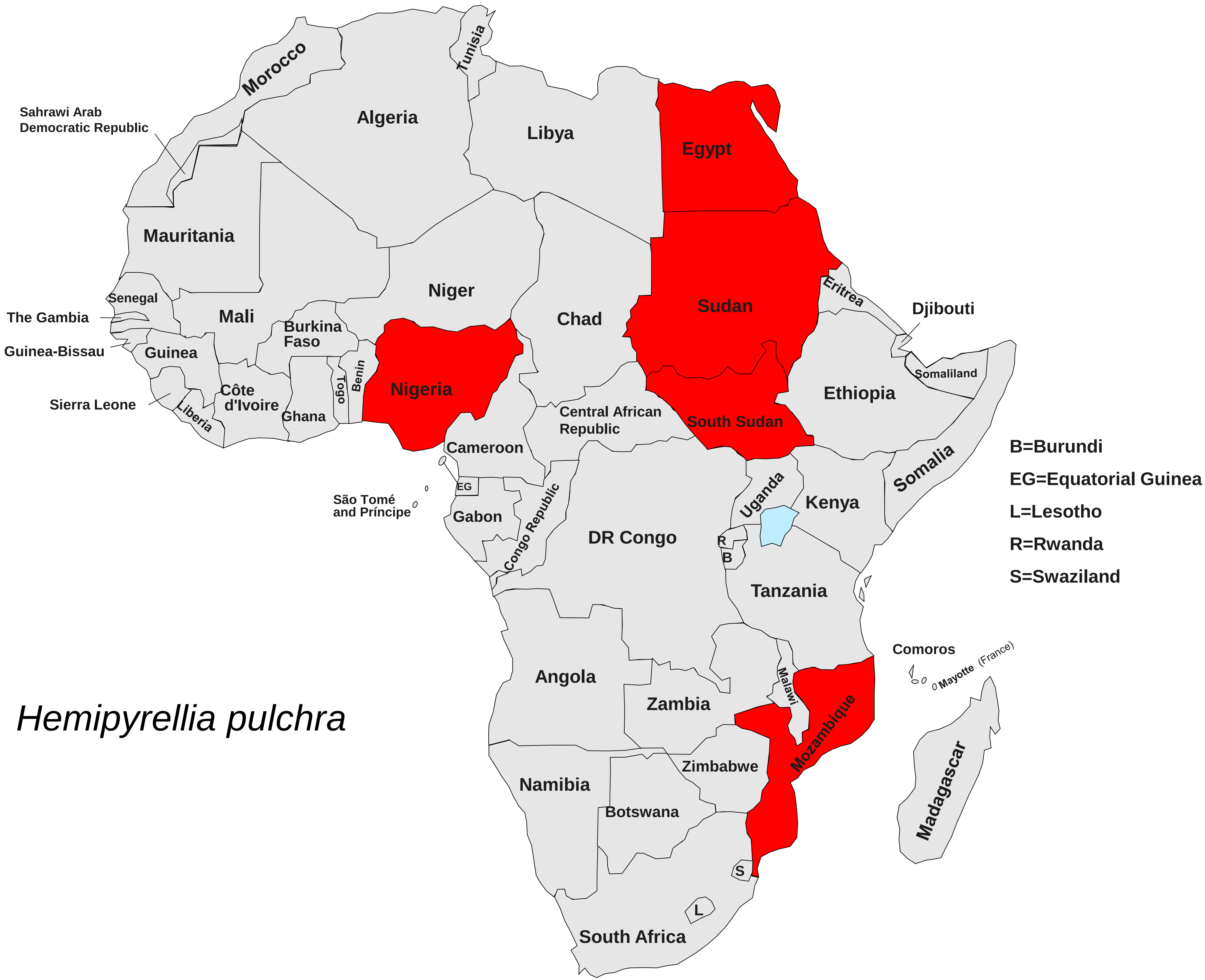

Hemipyrellia pulchra

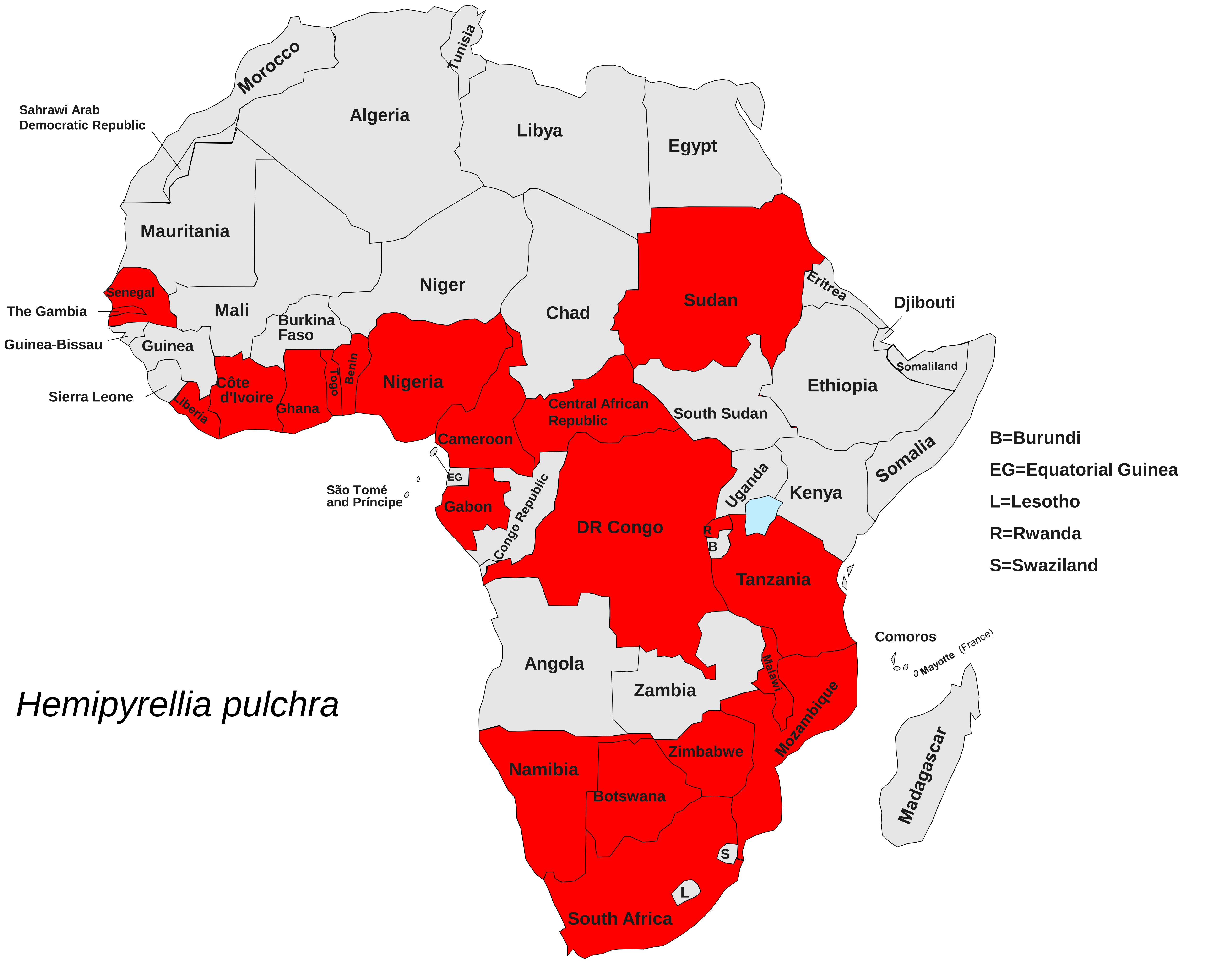

Hemipyrellia pulchra
